# Exploring the post-mortem interval through blood biochemistry: a preliminary case series study and review of the literature

**DOI:** 10.1007/s00414-025-03576-1

**Published:** 2025-08-19

**Authors:** Vincenzo M. Grassi, Gabriele Ciasca, Giuseppe Vetrugno, Andrea Urbani, Vincenzo L. Pascali, Fabio De-Giorgio

**Affiliations:** 1 Risk Management Unit, Fondazione Policlinico Universitario Agostino Gemelli IRCCS, L. go Agostino Gemelli 8, Roma, 00168 Italy; 2 Department of Healthcare Surveillance and Bioethics, section of Legal Medicine, Università Cattolica del Sacro Cuore, L.go Francesco Vito 1, Rome, 00168 Italy; 3 Dipartimento di Neuroscienze, Sezione di Fisica, Università Cattolica del Sacro Cuore, Rome, 00168 Italy; 4 Fondazione Policlinico Universitario Agostino Gemelli IRCCS, L. go Agostino Gemelli 8, Roma, 00168 Italy; 5 Department of Laboratory and Infectious Diseases Sciences, Fondazione Policlinico Universitario A. Gemelli IRCCS, Rome, Italy

**Keywords:** Post-mortem biochemistry, Post-mortem interval, Forensic pathology, Time of death

## Abstract

Post-mortem biochemistry can be included among the methods reported in the literature to investigate the time since death. To address this issue, we collected blood samples from hospitalized patients who died in the presence of health personnel. Samples were collected 20 min after death and every 6 h until 24 h post-mortem, with the last Ante Mortem (A.M.) sample also included in the analyses. Each sample was immediately centrifuged and analyzed. Our preliminary results indicate a significant and consistent increase in phosphokinase (CPK) and lactodehydrogenase (LDH) blood levels, with low interindividual variation among subjects. Interestingly, the measured time trend follows an exponential function, characterized by a time-dependent growth rate. While a larger sample size is needed to confirm our preliminary findings, our results suggest that CPK and LDH blood levels could be valuable parameters for determining the post-mortem interval. Furthermore, our data were systematically compared to those reported in the literature, which was carefully reviewed.

**Supplementary Information:**

The online version contains supplementary material available at 10.1007/s00414-025-03576-1.

## Introduction

Post-mortem biochemistry is commonly used to identify the physio-pathological changes involved in the death process and detect the presence of previous pathologies, such as diabetes, kidney or liver failure, etc., which may have played a significant role in determining death, especially in the absence of morphological changes detectable on autopsy examination and in cases of sudden death [1].

Several body fluids can be analyzed post-mortem: blood, CSF, vitreous humor, pericardial and synovial fluid. Each of these has its own characteristics. For example, changes occurring in the blood occur faster than vitreous humor [1]. Therefore, blood is more useful, in the immediate post-mortem interval, than vitreous humor, which provides more information when the time since death is longer than 24 h, also taking into account phenomena such as fluid redistribution or hemoconcentration [2, 3]. In addition, the sampling site is also relevant, with differences between peripheral blood and blood collected directly from the heart cavity [4].

The scientific literature points out a whole series of technical problems related to post- mortem biochemistry: the first concerns the temporal instability of analytes concentrations, which requires a standardization of collection timing. The second concerns the alteration of the tested fluids, when compared to the same analysis performed in clinical scenarios [1, 5], since machines and analysis methods are calibrated on samples taken from living subjects. It is also essential to establish a correlation between the values of a given analyte in an ante-mortem fluid and the values of the same analyte in the same post-mortem fluid. It is then necessary to determine whether, how and how quickly a balance is reached between the same fluids before and after death. The achievement of some reference ranges is based on three assumptions: the distribution of values in the population follows a Gaussian trend; samples are performed on a healthy population; samples are taken randomly. Such conditions are difficult to achieve, for example if samples are collected from hospitalized patients. Indeed, the reference ranges are affected by the population and its selection, the conditions of sample collection, the technique, and finally time of sampling, transport, preparation and storage of the sample withdrawn [6, 7]. In addition, the specific characteristics of the subject tested, such as age, sex, circadian rhythm, lifestyle, environmental influences, drug use, nutritional status, cause of death, duration of the agonic period, as well as post-mortem environmental conditions and sampling timing may influence the results of the analysis [8], thus requiring multicenter serial studies and standardized procedures [9].

Inspired by the diffuse use of virtopsy in the evaluation of the cause of death, which also gave rise to the term “toxopsy” [10], the purpose of the present study is to identify a biochemical marker to be used in the estimation of the time since death.

## Materials and methods

A total of three human cadavers were investigated in this study. All the subjects were selected from patients deceased during hospitalization. The inclusion criteria were the following: the death had to be occurred in presence of health personnel, therefore, the time of death had to be exactly known; a complete laboratory test must have been performed the same day prior to death. The exclusion criteria included patients deceased in Emergency Department and in Surgical Units, those affected by polytrauma and those who underwent cardiopulmonary resuscitation.

Case #1: 83-year-old male with history of invasive melanoma localized at the anterior thoracic wall, admitted for onset of dyspnea at rest; a CT scan detected multiple pleural localizations and massive pleural effusion. The cause of death was terminal respiratory failure.

Case #2: 67-year-old male affected by hepatocarcinoma and systemic hypertension. Admitted to our Policlinic for worsening hepatic failure, the cause of death was hepatic and kidney failure.

Case #3: 73-year-old female affected by cirrhosis in chronic hepatitis C infection, admitted to the hospital for decompensated ascites and anemia. The cause of death was respiratory failure.

A total of five sample has been collected form the same body. In accordance with the Italian law, a continuous 20-minute ECG was performed to ascertain the death before withdrawing the first peripheral blood sample from femoral vein; this was our time zero (T0) sample. Then, four further blood sample were taken from the same anatomic site every six hours, i.e., T1 + 6 h, T2 + 12 h, T3 + 18 h, T4 + 24 h. Each sample was transported under standard conditions to the Clinical Chemistry Laboratory, where it was immediately centrifuged at 4000 rpm for 5 min and analyzed using spectrophotometric technique [Olympus AU5400 automatic analyzer]. Additionally, the last blood sample Ante Morten (A.M.), was considered in our analysis. It should be mentioned that for each of the 15 samples obtained, we had to address the issue of hemolysis and its potential impact on the biochemical investigation [11]. Hemolysis can be attributed not only to the standardization of the sample collection and transport technique but also to the subject’s pathophysiology.

Data analysis and visualization was carried out with the software package R and OriginPro. We identified 5 missing values at random out of 342 analyte determinations. These values were imputed using the corresponding means calculated over the two remaining cases at the specific time. The presence of statistically significant differences among different times was assessed by using Anova for repeated measures.

## Results

In this paragraph, we describe the results of post-mortem blood biochemistry acquired from a case series of three cases.

**Fig. 1:**
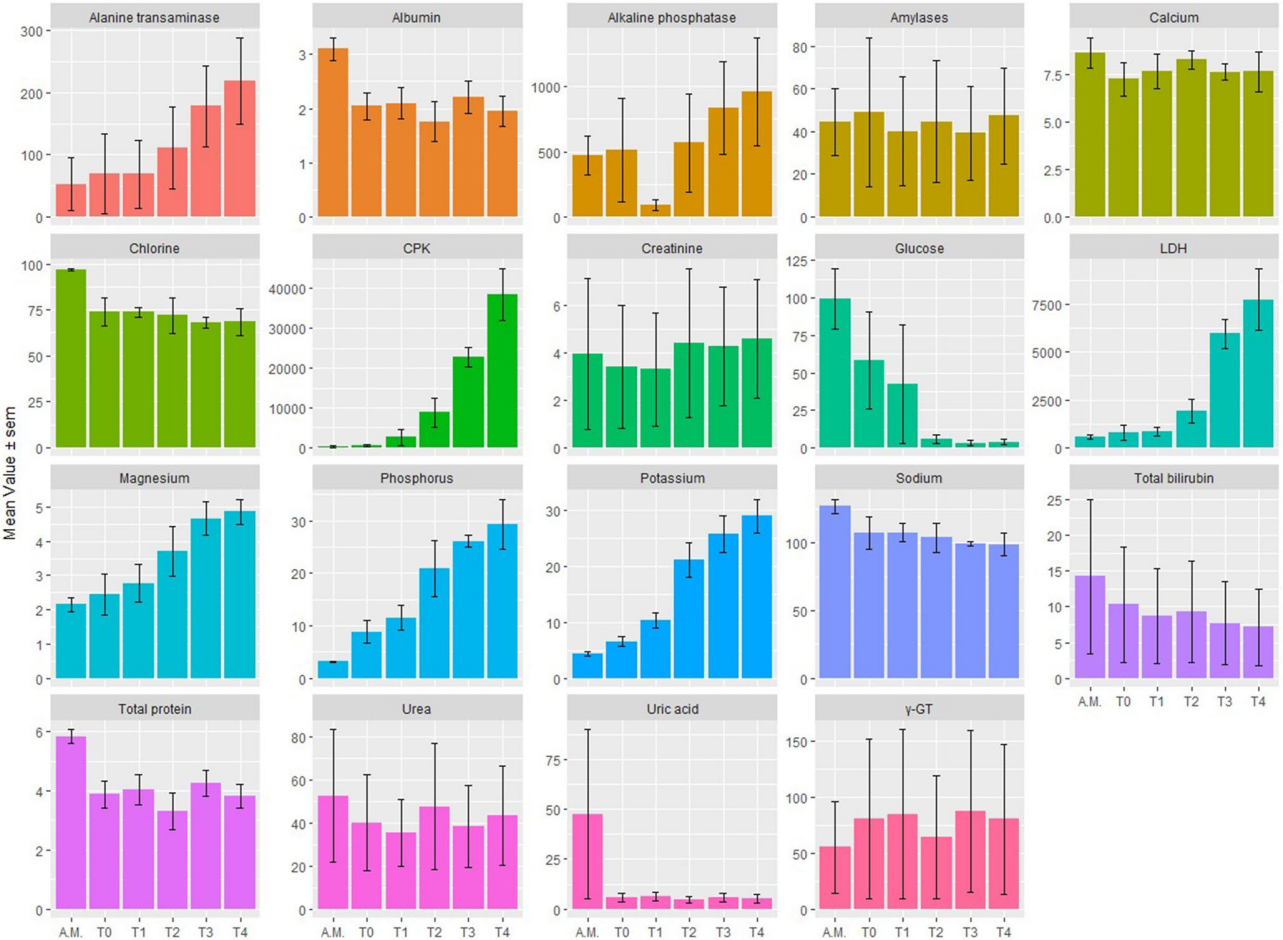
Time trend for each analyte measured Ante Mortem (A.M.), 20 min after death (T0), and every six hours. Data are reported in terms of mean ± SEM evaluated on the recruited corpses. Physical units are reported in Table S2, together with reference levels

Figure 1 displays the time trends for each analyte measured at Ante Mortem (A.M.), 20 min after death (T0), and every subsequent six hours, as described in the materials and methods section. The data are reported as mean ± SEM computed from the recruited corpses. The presence of statistically significant differences among time points was assessed using repeated measures ANOVA (Table 1). The complete set of analyte blood levels measured from the three corpses is reported in Table S1.

**Table 1:** Repeated measures ANOVA results

Analyte	DFn	DFd	F	*p*	*p* < 0.05	ges
Alanine transaminase	5	10	3.614	0.04	*	0.34
Albumin	5	10	5.984	0.008	*	0.526
Alkaline phosphatase	5	10	1.77	0.207		0.274
Amylases	5	10	0.172	0.967		0.009
Calcium	5	10	0.419	0.826		0.146
Chlorine	5	10	3.371	0.048	*	0.571
CPK	5	10	19.197	7.76E-05	*	0.898
Creatinine	5	10	2.006	0.163		0.016
Glucose	5	10	4.887	0.016	*	0.555
LDH	5	10	13.943	0.000309	*	0.863
Magnesium	5	10	14.046	0.000299	*	0.674
Phosphorus	5	10	13.324	0.000374	*	0.812
Potassium	5	10	17.327	0.000122	*	0.895
Sodium	5	10	1.422	0.297		0.403
Total bilirubin	5	10	1.63	0.239		0.046
Urea	5	10	1.007	0.461		0.029
Uric acid	5	10	1.07	0.431		0.289
γ-GT	5	10	0.92	0.506		0.016

A qualitative analysis of Fig. 1 suggests that several analytes in the blood exhibit monotonous time trends, either increasing or decreasing, which may be useful in determining the time since death. Specifically, an increasing trend is observed for Alanine Transaminase, CPK, LDH, Magnesium, Phosphorus, and Potassium, while a decreasing trend is observed for Glucose. The remaining variables appear to be constant over time. Consistent with these findings, the repeated measurements ANOVA in Table 1 reveals statistically significant differences in Alanine Transaminase (*p* = 0.04), Albumin (*p* = 0.008), Chlorine (*p* = 0.048), CPK (*p* = 7.76E-05), Glucose (*p* = 0.016), LDH (*p* = 0.00031), Magnesium (*p* = 0.00029), Phosphorus (*p* = 0.00037), and Potassium (*p* = 0.00012). Notably, significant analytes in Table 1 exhibit remarkable generalized η^2^ (GES) values. Despite the small sample size, the results presented here are generally consistent with previous literature, which will be discussed in detail in the next section, thus providing valuable information for forensic practitioners.

**Fig. 2:**
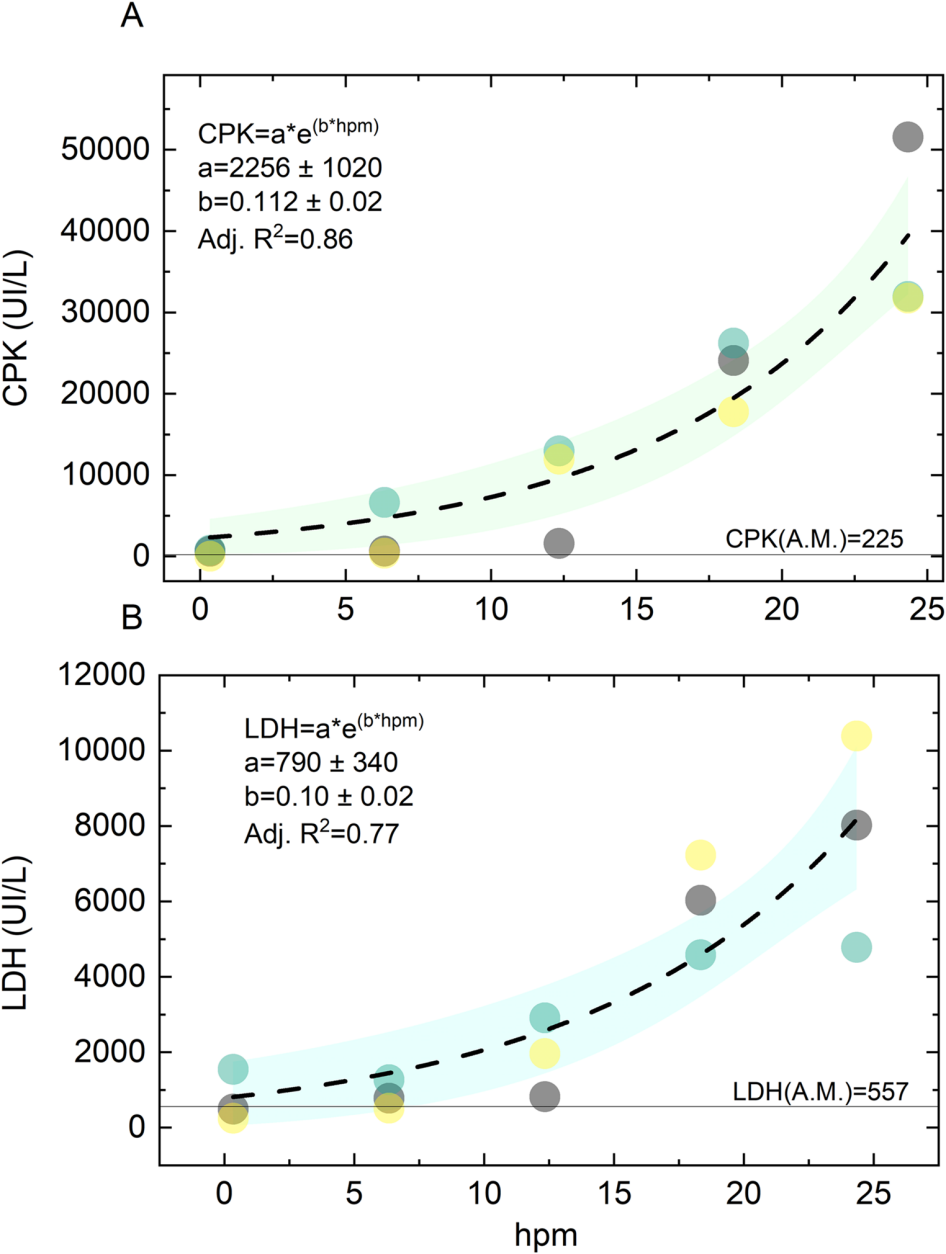
The time trend for CPK (**A**) and LDH (**B**) is shown, with data from different subjects represented using different colors. The ante mortem levels of the measured analytes are displayed as a continuous horizontal line. Each plot also includes the result of a non-linear fitting of the experimental points with an exponential function

In Fig. 2, we conduct a more detailed analysis of the analytes that have shown the highest significance, aiming to provide a more accurate description of their time-dependent changes. Among these biomarkers, CPK and LDH are particularly interesting as they exhibit a significant monotonous increase after death, following a non-linear trend. Compared to linear trends, we believe that non-linear ones may offer additional valuable information for determining the time of death. Linear trends have a constant rate of change, and thus, differential measurements taken at subsequent times do not provide any additional information in the presence of inter individual variability. In contrast, non-linear monotonous increases have a continuously varying growth rate that could be instrumental for determining the time of death when measured at adjacent time points. Interestingly enough, glucose also show a clear non-linear decrease. However, due to high inter-individual variability, as indicated by the extremely large SEM bars in Fig. 1, this analyte is not further investigated, as discussed in the next section. Additionally, Potassium also displays a potentially interesting non-linear increase. Nonetheless, it is not further investigated, as immediately after death, it escapes from the inside of the cells outwards, a phenomenon that complicates its analysis.

Accordingly, Fig. 2A and B show the CPK and LPH levels as a function of time, respectively. Data from different subjects are represented using different colors. A.M. levels of the measured analytes are displayed as a horizontal continuous line. In both cases, a qualitative observation of the investigated interval suggests an increasing rate of change of the biomarkers over time. Therefore, we decided to analyze the data using the exponential function $\:C\left(t\right)=\text{a}{e}^{bt}$, where “*a”* can be interpreted as the analyte concentration at the time of the death and “*b”* as the inverse of the exponential time constant. The best regression curves (black dashed lines) are superimposed to the experimental points together with the corresponding 95% confidence bands (shaded areas).

The fitting coefficients obtained were also displayed as an inset in the corresponding plot along with the adjusted R^2^ of the fit. It can be observed that in both cases, the experimental data points were well-fitted by the models with small confidence bands, despite the small sample size. Moreover, the adjusted R^2^ was remarkably high, indicating that a large portion of the experimental variability was explained by the model. Overall, the results presented in Fig. 2 suggest that the exponential formula with the retrieved coefficients could be useful for estimating the time of death, although it needs to be tested and adjusted on a larger sample size.

However, it is important to note that we observed a strong and statistically significant positive correlation (Pearson’s coefficient ρ = 0.86, *P* < 0.000) between CPK and LDH levels. Therefore, the two parameters should not be combined in a multivariate analysis to predict the time of death. Instead, the most informative marker should be selected. Based on our preliminary data, we recommend using CPK due to its higher adjusted R^2^.

## Discussion

In this section we aim to provide the reader with a concise but informative literary review on blood post mortem biochemistry, organized in bullet points, focused on the different metabolites. This literature review is systematically compared with our finding to highlight possible applications in the determination of the time since death.

The evaluation of the time since death represents one of the most challenging objective in everyday forensic practice and, therefore, a topic of great relevance in scientific research. Apart from the classical temperature-method calculation, new experimental approaches have been recently proposed [12–14].

Postmortem biochemistry has been classically applied in determining the cause of death [1, 15, 16]. Later, different biochemical methodologies for the determination of the post-mortem interval have been developed, mainly focused on vitreous humor [17–20], before evolving towards proteomic and metabolomic approaches [21, 22].

An ideal biochemical marker should present the following characteristics: rapid response at the end of the vital processes; post-mortem stability; high sensitivity and specificity; sufficient amount; easy accessibility; low costs and quick analysis; standardization and quality certification. Maeda et al. [9] classify biochemical markers in three groups: stable and comparable to clinical criteria; - predictable agonal/postmortem interference; - unpredictable agonal/postmortem interference.

Coe has been the author of numerous publications on postmortem biochemistry since the late 1960 s [23–28] and has laid the foundations for numerous subsequent studies.

Scientific literature, mainly focused on the cause of death, provides the following data regarding post-mortem trend of the main analytes.

### Glucose

Samples taken in the right heart cavity or lower vena cava show elevated glucose levels, due to hepatic glycogenolysis and subsequent glucose diffusion in the bloodstream. On the other hand, low glucose levels in these sites indicate, especially when combined with a positive test for ketone bodies, a situation of prolonged fasting. Elevated glucose values may also be obtained in peripheral blood samples, particularly if the cause of death is asphyxiation, cerebral hemorrhage, cardiac arrest, electrocution [27], as well as in subjects undergoing cardiopulmonary resuscitation [29], due to the release of catecholamines in the stages of terminal stress. A diagnosis of ante-mortem hyperglycemia should include the determination of glycated hemoglobin (HbA1c) and fructoosamine [30, 31]. For the evaluation of hyperglycemia, vitreous humor has become the reference sample, with a sudden decrease in glucose concentration in normal subjects, while in diabetic patients this decrease is slower and less evident [32]. In vitreous humor, glycolysis is slowed by the cooling of the corpse immediately after death [33], while glucose levels are higher in subjects dying of hypothermia [34], due to terminal stress-induced hyperglycemia.

As expected, a similar stable decrease in sugar levels, likely due to red blood cell glycolysis, is observed in Fig. 1. Interestingly, this decrease appears to be non-linear, with an initial fast decay followed by a saturation phase where negligible sugar concentrations are measured. In our data, this saturation phase can be observed at 12 h post-meal (hpm). As previously explained, we believe that non-linear trends with saturation phases can be used to determine the time of death either prior to or after a given threshold time. However, to accomplish this, it is necessary to account for potential confounding factors, such as diabetes, which contribute to high variability among subjects.

### Electrolytes

#### Sodium

As demonstrated by Coe [25], blood sodium levels begin to decrease immediately after death, at an average rate of 0.9 meq/L; however there are inter-individual variations that do not allow the use of this electrolyte to determine the post-mortem range. Instead, vitreous humor showed relatively stable sodium levels during the early post-mortem period [35]. The results in Fig. 1 support Coe’s findings on blood, which showed an abrupt decrease immediately after death, followed by a slower steady decrease at subsequent times. It is noticeable that the standard error of the mean (SEM) values are large compared to the changes in sodium levels. This variability is likely due to the individual differences among subjects, as suggested by Coe, which may have resulted in non-significant results in Table 1.

#### Chlorine

Several authors have described a decrease in chlorine after death [25, 36]. Coe [27] determined that the rate of decrease of blood levels of chlorine is on average 0.97 meq/L/h. Nevertheless, even in the case of chlorine, there are inter-individual variations which do not allow this electrolyte to be used to determine the post-mortem range. Again, our results support this finding, as a post-hoc analysis of chlorine data does not show any significant difference between T0 and T4. Conversely, the slight significance observed in Table 1 is entirely dependent on the A.M. level of chlorine. Similarly to sodium, also for chlorine in vitreous humor it is possible to correlate changes in lifetime concentrations with post-mortem values, since normal values (115–125 meq/L) [23, 37], show only a slight decrease in the early post-mortem period.

#### Potassium

Immediately after death, potassium begins to escape from the inside of the cells outwards, thus causing an increase in the concentration of potassium both in the blood and in the vitreous humor. This is also clearly shown by our data in Fig. 1. This phenomenon makes it almost impossible to use post-mortem blood potassium concentrations to establish ante-mortem alterations of this electrolyte [38, 39]. The increase in potassium vitreal concentration occurs more slowly and linearly; this led some authors to focus on this parameter for the estimation of the post-mortem interval [40, 41]. Several authors have developed their own equation to determine the time of death, including Madea [42], whose equation «PMI (h) = 5.26 x [K+] (mmol/L) − 30.9» is one of the most appreciated and used. The rate of increases is influenced by internal factors, including death from chronic disease and uremia [6], and external factors, such as body cooling.

#### Calcium

The immediate post-mortem measurement of calcium concentration in the blood yielded different results according to the analytical methods used [36], showing constant values, as also occurred in our case, or a time-related increase. A rather slow increase in vitreous occurs [43], which begins immediately after death and ends with decomposition [41]. Moreover, the levels of calcium in the vitreous humor are not correlated to ante-mortem blood levels, so that, in cases of known hypocalcemia in-vitam, calcium levels of humor vitreous were normal [44].

#### Magnesium

According to Jetter, the blood concentration of magnesium remains low until hemolysis occurs, which leads to the release of magnesium outside the cells, resulting in an increase in the blood concentration of magnesium, up to levels of 20–30 meq/L [36]. Coe subsequently demonstrated an early and progressive increase in the blood concentration of magnesium [25], which is also confirmed by our data. A similar increase has been demonstrated in vitreous humor, where a slow increase in concentration has been identified, related to the increase in the postmortem interval, but with a such variability to make it unreliable for the evaluation of the time since death [41]. Furthermore, also in vitreous humor, a progressive increase in the concentration of magnesium in cadavers of subjects drowned in salt water has been described, thus providing the possibility of determining how long a cadaver has remained immersed [45].

#### Phosphorus

The study by Jetter [36] reported an increase in serum concentrations of organic and inorganic phosphorus. This result is confirmed by the data shown in Fig. 1. Inorganic phosphorus begins to increase an hour after death and reaches concentrations of 20 meq/L eighteen hours after death. Finally, in the vitreous humor, the average concentration of inorganic phosphorus is 1.2 meq/L.

### Total protein and albumin

The post-mortem blood values of total proteins are kept within the reference ranges of living [27], while the electrophoretic pattern shows differences only in the case of significant hemolysis. After death, there is a 4% decrease in albumin values and a 5% increase in β globulin, while the other fractions remain substantially unchanged.

In vitreous humor protein values range between 40 and 80 mg/dl. The younger the subject, the closer the values to the lower limits; on the contrary, the older the subject, the closer the values to the upper limits. In vitreous humor, high levels of glycoproteins account for qualitative differences between this fluid and blood. Moreover, while all the proteins present in the blood can be found, even if in traces, in the posterior chamber of the eye, there are some proteins that are present only in the vitreous humor.

### Nitrogen compounds

#### Urea

Post-mortem blood levels of urea are similar to ante-mortem levels and are independent of the post-mortem interval, the method of analysis used, the urea levels found and even decomposition. Our data provide strong confirmation of this finding, showing that urea levels are kept remarkably constant over time (Fig. 1). In hospitalized patients, who died in the absence of kidney disease, the average of measurements was 47.4 mg/dl, while in those who died suddenly, urea levels ranged between 13 and 15.5 mg/dl [28]. In the vitreous humor, a slight increase in urea after death has been demonstrated, however post-mortem values are overlapping with ante-mortem values [37]. Coe states that urea is the most stable compound among all elements considered in post mortem [23]. In fact, in over 90% of cases, the author found a variation of less than 3 mg/dl in blood samples taken up to 100 h after death. In vitreous, for urea values below 100 mg/dl, a correlation with the post-mortem interval can be established, with a 95% confidence interval up to 120 h after death of 21.78 h [46]. Values above 100 mg/dl indicate lifetime urea retention related to chronic diseases.

#### Creatinine

Like urea, blood creatinine is quite stable after death and independent of the time of sampling. Regarding vitreous humor, the values found, although slightly lower, correspond to the blood values. Determination of creatinine levels can be used to determine urea retention. If creatinine values in vitreous humor are less than 1 mg/dl, in combination with urea values in vitreous less than 70 mg/dl, the postmortem interval can be estimated with an error of 15 h, with a 95% confidence interval up to 120 h after death [46].

#### Uric acid

Serum uric acid levels range from 5.5 to 6.2 mg/dl between six and eight hours after death. In vitreous humor, uric acid has an average concentration of 1.3 mg/dl four hours after death and 1.5 mg/dl sixteen hours after death [43].

### Bilirubin

Analysis of the blood bilirubin concentration showed a slight but steady increase after death, quantifiable in 0.2 mg/dl after two hours and in 0.7 mg/dl after twenty hours [25]. However, in jaundice subjects, this increase is so slight that it is masked by high starting levels. Small amounts of bilirubin were also found in vitreous humor, with a ratio of 220:1 referred to blood [47].

### Enzymes

Transaminases, amylases, creatine phosphokinase, lactodehydrogenase, pseudocholinesterase, γ-GT and alkaline phosphatase.

Studies carried out by Naumann [48], Enticknap [49] and Hall [50] showed a post-mortem increase in serum concentrations of alkaline phosphatase, amylase, transaminase, lactodehydrogenase and creatine phosphokinase. This increase occurs rapidly after death and in an unpredictable way, so the post-mortem determination of these enzymes is of little value. The concentration of acetylcholinesterase remains stable after death, since no significant changes occur from ante-mortem values up to three weeks after death [51]. Total cholinesterase, given by the sum of acetylcholinesterase and pseudocholinesterase, could be used in the diagnosis of the time of death, but interim variations, related to the duration of the agonic period, make this enzyme unsuitable for this purpose. Studies on γ-GT have established a correlation between high postmortem serum levels of this enzyme and chronic alcohol abuse, which is also evident in living animals [52, 53]. In the vitreous humor, after initial studies which failed to measure enzymes [37], transaminases and LDH were subsequently dosed, but at minimal and extremely variable concentrations, as well as independent of ante-mortem values or previous pathologies. γ-GT is able to flow from the blood to the vitreous humor in vitro, crossing the barrier that separates these two fluids [54]. Amylase also penetrates the eyeball and a slight increase in pancreatic amylase in deaths due to acute hypothermia and an increase in isoamylase in cases of prolonged hypothermia has been shown [55].

In summary, our results show that glucose concentration exhibited a constant decrease, but this trend was affected by high variability among subjects, rendering it unsuitable for estimating the time since death. However, it is noteworthy that glucose levels were found to be close to zero from the 12th hour even in cases where the ante-mortem values were higher than the upper limit of the reference range. This interesting threshold can be used to determine the time of death either before (if saturation is not reached) or after (if saturation is reached) the 12th hour.

The results of the electrolyte analysis (Na, K, Cl, Ca, Mg, P) are particularly susceptible to alteration due to hemolysis. In our study, the measured concentrations exhibited an irregular alternating profile of increase and decrease among patients (as shown in Table S1), making the results unreliable for assessing the time since death. Only in samples with minimal hemolysis, an increase in potassium was observed, which was expected based on previous literature. However, our results did not allow us to establish a valid correlation between potassium levels and the time since death due to the interference of the hemolytic process. Both total proteins and albumin have not shown a uniform behavior, such as to be considered for the diagnosis of the time since death. Nitrogen compounds and bilirubin also showed an irregular pattern.

Both CPK and LDH exhibit a significant and steady increase after death, as shown in Fig. 2. Interestingly, the experimental data points can be accurately described by an exponential function over time intervals. This mathematical trend, computed with the retrieved fitting coefficient, has potential for use in determining the time since death and estimating its error. Notably, this increase has a solid biological basis, as both CPK and LDH are considered markers of cellular necrosis in living systems. Moreover, this elevation is also observed in cases of bodily incisions during life.

Transaminases, amylases, pseudocholinesterases, γ-GT and alkaline phosphatases presented an irregular blood concentration profile, as described in the literature [48–50]. Transaminases, markers of hepatocellular necrosis in life, have not shown an increase, in accordance with the resistance of this organ to ischemia, as known from organ transplant studies. This behavior is not verifiable from our data, although early and steady increases have been noted in samples that are poorly contaminated by hemolysis. The expected decrease of pseudoChE has not occurred; indeed, in one case there has been a constant increase, but never exceeding the lower limit of the reference range in the living. Finally, the expected increases, according to literature [48–50], of γ-GT and FA have not been consistently confirmed.

## Study limitations

The limitation of this study lies in the small number of samples analyzed, partially related to the inclusion/exclusion criteria adopted, therefore the present contribution has to be certainly considered preliminary.

Another limitation derives from the well-known pracantemortemculties of obtaining post-mortem human blood samples, which led other Authors to focus on animal sampling [56, 57].

Finally, hemolysis greatly influences the results of laboratory analysis and the more the length of the post-mortem interval increases, the more this phenomenon is relevant.

## Conclusions

This preliminary study highlights the importance of identifying a post-mortem biochemistry marker that can be useful applied when estimating the time since death. According to our preliminary evaluation, the most encouraging analytes are creatine phosphokinase and lactodehydrogenase, which showed a significant and constant increase in their concentrations after death, following an exponential behaviour. Compared to previous studies [49], our preliminary results suggest that the increase in these enzymes is not unpredictable and of little use in post-mortem biochemistry [58]; however, further confirmation is needed, obtainable through the extension of this case study, to establish a valid correlation between a specific value of one of these two enzymes and the post-mortem interval.

## Supplementary Information

Below is the link to the electronic supplementary material.

Supplementary Material 1

## Data Availability

The report does not contain personal data. In any case, all data are covered by the Italian Law—Data Protection Authority (Official Gazette no. 72 of March 26, 2012)—for scientific research purposes.
