# Supplementary material for: Exploring the post-mortem interval through blood biochemistry: a preliminary case series study and review of the literature
Source: Int J Legal Med. 2025 Aug 19;139(6):3093–102. doi: 10.1007/s00414-025-03576-1 (PMC12532683; doi:10.1007/s00414-025-03576-1)
Supplement: Supplementary file 1 — Supplementary Material 1 [file 414_2025_3576_MOESM1_ESM.docx]

**Appendix A**

| Analyte | case | A.M. | T0 | T1 | T2 | T3 | T4 |
| --- | --- | --- | --- | --- | --- | --- | --- |
| **Alanine transaminase** | **A** | 6 | 3 | 5.2 | 6.3 | 61 | 99.2 |
| **Alanine transaminase** | **B** | 139 | 197.1 | 178.5 | 232 | 285.2 | 219 |
| **Alanine transaminase** | **C** | 14 | 8.1 | 23.3 | 95 | 189.4 | 339.2 |
| **Albumin** | **A** | 3.5 | 2.54 | 2.69 | 1.9 | 2.64 | 2 |
| **Albumin** | **B** | 2.8 | 1.88 | 1.76 | 1.07 | 1.64 | 1.46 |
| **Albumin** | **C** | 3 | 1.71 | 1.85 | 2.31 | 2.36 | 2.42 |
| **Alkaline phosphatase** | **A** | 210 | 109 | 123 | 99 | 131 | 138 |
| **Alkaline phosphatase** | **B** | 729 | 1303 | 15 | 1311 | 1205 | 1285 |
| **Alkaline phosphatase** | **C** | 469.5 | 124 | 136 | 293 | 1172 | 1459 |
| **Amylases** | **A** | 17 | 15 | 13 | 9 | 5 | 6 |
| **Amylases** | **B** | 72 | 119 | 91 | 101 | 80 | 83 |
| **Amylases** | **C** | 44.5 | 13 | 16 | 24 | 33 | 53 |
| **Calcium** | **A** | 8.9 | 9 | 9.46 | 7.3 | 8.29 | 6 |
| **Calcium** | **B** | 7.2 | 6.65 | 6.34 | 8.72 | 6.88 | 7.32 |
| **Calcium** | **C** | 9.9 | 6.16 | 7.26 | 8.83 | 7.77 | 9.65 |
| **Chlorine** | **A** | 97 | 78 | 79 | 53 | 62 | 54 |
| **Chlorine** | **B** | 96 | 85 | 71 | 84 | 71 | 74 |
| **Chlorine** | **C** | 98 | 59 | 71 | 79 | 71 | 77 |
| **CPK** | **A** | 598 | 651 | 663 | 1616 | 24113 | 51579 |
| **CPK** | **B** | 57 | 772 | 6652 | 12996 | 26243 | 31986 |
| **CPK** | **C** | 20 | 46 | 420 | 11974 | 17817 | 31774 |
| **Creatinine** | **A** | 0.7 | 1.03 | 1.02 | 1.03 | 1.69 | 1.76 |
| **Creatinine** | **B** | 10.4 | 8.64 | 8.11 | 10.73 | 9.27 | 9.62 |
| **Creatinine** | **C** | 0.8 | 0.62 | 0.82 | 1.55 | 1.87 | 2.47 |
| **Glucose** | **A** | 137 | 122 | 122 | 11 | 3 | 3 |
| **Glucose** | **B** | 68 | 17 | 0 | 1 | 0 | 1 |
| **Glucose** | **C** | 94 | 35 | 6 | 6 | 6 | 7 |
| **LDH** | **A** | 542 | 495 | 784 | 823 | 6033 | 8026 |
| **LDH** | **B** | 755 | 1549 | 1275 | 2913 | 4588 | 4785 |
| **LDH** | **C** | 375 | 253 | 521 | 1966 | 7233 | 10392 |
| **Magnesium** | **A** | 2.15 | 2.82 | 2.69 | 3.02 | 5.49 | 4.88 |
| **Magnesium** | **B** | 2.5 | 3.24 | 3.77 | 5.15 | 4.72 | 5.49 |
| **Magnesium** | **C** | 1.8 | 1.26 | 1.86 | 2.94 | 3.77 | 4.21 |
| **Phosphorus** | **A** | 3.3 | 11.32 | 11.74 | 14.04 | 25.75 | 19.76 |
| **Phosphorus** | **B** | 3 | 10.55 | 15.58 | 31.38 | 28.16 | 34.01 |
| **Phosphorus** | **C** | 3.15 | 4.75 | 7.29 | 17.44 | 24.44 | 34.22 |
| **Potassium** | **A** | 3.8 | 8 | 8.8 | 16 | 32 | 32.2 |
| **Potassium** | **B** | 5.2 | 6.5 | 13 | 26.8 | 21.2 | 23 |
| **Potassium** | **C** | 4.1 | 5 | 9 | 20.6 | 23.7 | 31.7 |
| **Sodium** | **A** | 118 | 122 | 121 | 82 | 97 | 84 |
| **Sodium** | **B** | 128 | 117 | 101 | 115 | 99 | 101 |
| **Sodium** | **C** | 136 | 84 | 101 | 115 | 102 | 112 |
| **Total bilirubin** | **A** | 1 | 0.8 | 0.93 | 0.6 | 0 | 0.03 |
| **Total bilirubin** | **B** | 35.69 | 26.3 | 21.8 | 23.4 | 19.2 | 17.6 |
| **Total bilirubin** | **C** | 6.07 | 3.9 | 3.4 | 4 | 4 | 3.9 |
| **Total protein** | **A** | 6.3 | 4.73 | 5.06 | 3.51 | 4.98 | 3.79 |
| **Total protein** | **B** | 5.6 | 3.77 | 3.65 | 2.16 | 3.48 | 3.15 |
| **Total protein** | **C** | 5.6 | 3.17 | 3.39 | 4.29 | 4.32 | 4.55 |
| **Urea** | **A** | 21 | 22 | 24.6 | 17.9 | 20.6 | 14.3 |
| **Urea** | **B** | 114 | 84.3 | 65.9 | 106.1 | 76.7 | 89 |
| **Urea** | **C** | 23 | 13.7 | 15.9 | 19.1 | 18.4 | 26.8 |
| **Uric acid** | **A** | 2.5 | 2.88 | 2.95 | 1.81 | 2.06 | 1.64 |
| **Uric acid** | **B** | 132 | 9.9 | 9.98 | 6.79 | 9.56 | 8.44 |
| **Uric acid** | **C** | 8.1 | 4.8 | 5.65 | 5.91 | 5.46 | 5.75 |
| **γ-GT** | **A** | 15 | 10 | 10 | 7 | 14 | 11 |
| **γ-GT** | **B** | 136 | 223 | 235 | 174 | 231 | 214 |
| **γ-GT** | **C** | 14 | 8 | 9 | 11 | 16 | 16 |

**Table S1.** full record of blood levels of the investigated analytes for the 3 cases at the different time points, namely A.M. = ante-mortem. Var.= variation, in relation to reference ranges for A.M. and previous results for the post-mortem samples. T0 = sample withdrawn 20 minutes after the death. T1 = sample withdrawn at the time of death + 6 hours. T2 = sample withdrawn at the time of death + 12 hours. T3 = sample withdrawn at the time of death + 18 hours. T4 = sample withdrawn at the time of death + 24 hours.

**Table S2:** Units of measure and reference ranges in use at our laboratory.

| Parameter | Units of measure | reference ranges |
| --- | --- | --- |
| **Glucose** | mg/dl | 65–110 |
| **Sodium** | mEq/L | 135–145 |
| **Chlorine** | mEq/L | 98–108 |
| **Potassium** | mEq/L | 3.5–5.0 |
| **Calcium** | mg/dl | 8.50–10.50 |
| **Magnesium** | mg/dl | 1.80–2.40 |
| **Phosphorus** | mg/dl | 2.50–4.50 |
| **Total protein** | g/dl | 6.50–8.50 |
| **Albumin** | g/dl | 3.40–4.80 |
| **Urea** | mg/dl | 10.0–23.0 |
| **Creatinine** | mg/dl | 0.66–1.20 |
| **Uric acid** | mg/dl | 3.50–7.00 |
| **Total bilirubin** | mg/dl | 0.30–1.20 |
| **Aspartate transaminase** | UI/L | 7.0–45.0 |
| **Alanine transaminase** | UI/L | 7.0–45.0 |
| **Amylases** | UI/L | 5–100 |
| **CPK** | UI/L | 30–170 |
| **LDH** | UI/L | 230–460 |
| **PseudoChE** | UI/L | 5300–13000 |
| **γ-GT** | UI/L | 5–55 |
| **Alkaline phosphatase** | UI/L | 98–279 |

Mattedi RL, Bernardi F del C, Bacchi CE, et al (2007) Fatal outcome in bronchus-associated lymphoid tissue lymphoma. Jornal Brasileiro de Pneumologia 33:487–491
